# Machine learning-assisted prediction of pneumonia based on non-invasive measures

**DOI:** 10.3389/fpubh.2022.938801

**Published:** 2022-07-28

**Authors:** Clement Yaw Effah, Ruoqi Miao, Emmanuel Kwateng Drokow, Clement Agboyibor, Ruiping Qiao, Yongjun Wu, Lijun Miao, Yanbin Wang

**Affiliations:** ^1^College of Public Health, Zhengzhou University, Zhengzhou, China; ^2^Department of Radiation Oncology, Zhengzhou University People's Hospital, Henan Provincial People's Hospital, Zhengzhou, China; ^3^School of Pharmaceutical Sciences, Zhengzhou University, Zhengzhou, China; ^4^Department of Respiratory and Critical Care Medicine, The First Affiliated Hospital of Zhengzhou University, Zhengzhou, China; ^5^Center of Health Management, General Hospital of Anyang Iron and Steel Group Co., Ltd, Anyang, China

**Keywords:** pneumonia, machine learning, non-invasive measures, electronic health records (EHR), decision support system (DSS)

## Abstract

**Background:**

Pneumonia is an infection of the lungs that is characterized by high morbidity and mortality. The use of machine learning systems to detect respiratory diseases *via* non-invasive measures such as physical and laboratory parameters is gaining momentum and has been proposed to decrease diagnostic uncertainty associated with bacterial pneumonia. Herein, this study conducted several experiments using eight machine learning models to predict pneumonia based on biomarkers, laboratory parameters, and physical features.

**Methods:**

We perform machine-learning analysis on 535 different patients, each with 45 features. Data normalization to rescale all real-valued features was performed. Since it is a binary problem, we categorized each patient into one class at a time. We designed three experiments to evaluate the models: (1) feature selection techniques to select appropriate features for the models, (2) experiments on the imbalanced original dataset, and (3) experiments on the SMOTE data. We then compared eight machine learning models to evaluate their effectiveness in predicting pneumonia

**Results:**

Biomarkers such as C-reactive protein and procalcitonin demonstrated the most significant discriminating power. Ensemble machine learning models such as RF (accuracy = 92.0%, precision = 91.3%, recall = 96.0%, f1-Score = 93.6%) and XGBoost (accuracy = 90.8%, precision = 92.6%, recall = 92.3%, f1-score = 92.4%) achieved the highest performance accuracy on the original dataset with AUCs of 0.96 and 0.97, respectively. On the SMOTE dataset, RF and XGBoost achieved the highest prediction results with f1-scores of 92.0 and 91.2%, respectively. Also, AUC of 0.97 was achieved for both RF and XGBoost models.

**Conclusions:**

Our models showed that in the diagnosis of pneumonia, individual clinical history, laboratory indicators, and symptoms do not have adequate discriminatory power. We can also conclude that the ensemble ML models performed better in this study.

## Introduction

Pneumonia has been a major cause of morbidity and mortality in both developed and developing countries, especially among patients who are diagnosed and treated at a later stage (1, 2). Specific symptoms such as cough with sputum production, fever, chest pain, shortness of breath, and chills are the main characteristics associated with pneumonia (3). Because of several reasons such as difficulty in identifying the etiological agents in individuals, low specificity of symptoms of lower respiratory tract infections, and lack of widespread availability of laboratory tests and imaging, the accurate definition and diagnosis of pneumonia are still debatable (4). Diagnostic findings such as decreased breathing sounds, crackles, bronchial breath sounds, egophony, along with a sharp increase in body temperature, tachypnea, hypoxia, tachycardia, and dyspnea, should suggest pneumonia (either broncho- or lobar). Pneumonia benefits from antibiotics. So, to prevent unnecessary administration of antibiotics that might ultimately create multi-drug-resistant “superbugs” - as has already happened - procalcitonin levels are monitored along with clinical symptoms. Procalcitonin is released from lung neuroendocrine cells after exposure to bacterial endotoxin and lipopolysaccharides which typically increases the production of procalcitonin. The appearance of pneumonia symptomatology coupled by a rise in procalcitonin levels would trigger antibiotic administration.

Although chest radiography is the recommended technique for pneumonia diagnosis, factors such as lack of standardized interpretation (5), inter-rater variability (6), absence of abnormalities in the chest radiographs of children (7), low sensitivity to early-stage pneumonia, and potential harm due to exposure to x-rays hinder their use. Most importantly, radiography is usually not available in areas with the highest disease burden such as those in low-income settings. Consequently, general practitioners mainly rely on non-invasive measures such as the use of signs, symptoms, and simple laboratory tests as tools to diagnose pneumonia. To improve diagnostic accuracy and enhance various treatment strategies for pneumonia, prediction models based on non-invasive measures have been proposed.

Machine learning (ML), a powerful computer-based method that has the capacity to learn, reason, and self-correct without explicit programming, has the potential to provide solutions to the above problems. In recent years, the use of ML has achieved great advances and major benefits in medicine. Researchers have used large clinical databases to answer previously unanswerable questions and create systems that facilitate human decision-making (8, 9). Over the years, enthusiasm and optimism have been alternated with skepticism and pessimism in this fascinating field of research. Although some claims associated with this kind of research are currently being made with great grandiose claims (10), ML-based models have already proven to be useful in some clinical applications (11). ML has been shown to improve diagnostic accuracy for pneumonia when applied to hospitalized patients (12). The use of machine learning techniques to detect pneumonia *via* non-invasive measures such as signs and symptoms is gaining much attention. In several clinical studies, clinical history and physical examination parameters have been evaluated for their diagnostic value in predicting pneumonia (13, 14).

Based on the above, this study conducted several experiments on various ML models to predict pneumonia based on biomarkers, laboratory, and physical features.

## Methods

### Data collection and preprocessing

We retrospectively recruited patients aged at least 18 with confirmed acute lower respiratory illness and treated at the First Affiliated Hospital of Zhengzhou University in Henan Province between October 29, 2019, and May 21, 2021. The First Affiliated Hospital of Zhengzhou University is one of the largest hospitals in central China, with an ~13,000-bed capacity. We extracted patient demographic information (including age, sex, and comorbidities), physical parameters (tachycardia, tracheal secretion, pleural effusion, mean arterial pressure, heart rates, breathing rates, and systolic blood pressure), and hematological parameters. Hematological parameters included serum sodium, serum potassium, serum creatine, hematocrit, WBC count, platelet, total bilirubin, hemoglobin, C-reactive protein (CRP), and procalcitonin (PCT). Unfortunately, some patients had some missing data. As a result, we later addressed some of these missing values in the dataset (data preprocessing). Typically, real-world data contains multiple errors, incompleteness, and incoherence, requiring data preprocessing. Because of this, we preprocessed the data following these four steps:

#### Missing values

Missing data causes problems when a ML model is applied to the dataset. Mostly, ML models don't process data with missing values. In this study, some variables had several missing values of about 15% of that variable data. We used the median and mode of the corresponding columns to fill in the missing values of numerical attributes and categorical attributes, respectively. Median is the centrally located value of the dataset in ascending order. We filled missing numerical attribute values with the median value. Mode is the most repeated value in the given categorical observations. We filled missing entries with the mode observations.

#### Imbalance data

The dataset was unbalanced. A classification dataset with skewed class proportions is called imbalanced. Classes that make up a large proportion of the dataset are called majority classes. Those that make up a smaller proportion are minority classes. The degree of imbalance in the minority class can be mild (20–40% of the dataset), moderate (1–20% of the dataset), and extreme (<1% of the dataset). In this study, the minority class was 22% lesser than the majority class. Therefore, we needed to resolve the issue before applying machine learning in order to reduce data bias. One of the over-sampling approaches to fix imbalanced data is the synthetic minority over-sampling technique (SMOTE) (15). It manages overfitting induced by a limited decision interval by controlling the generation and distribution of manual samples using the minority class sample. Specifically, SMOTE is based on selecting a random minority class as the last sample. Then it finds the *k* nearest neighbors (normally *k* = 5) of the selected prior sample. Finally, it selects a random neighbor and creates a synthetic sample between the two samples (prior sample and selected neighbor) in the feature space at a randomly selected point. We can express SMOTE as


$$\begin{array}{l} SMOTE\left(x_{syn}\right)=x_{p}+\left(x_{knn}-x_{p}\right)\times \;\alpha , \end{array}$$

where *x*_*p*_ denotes feature vector of a prior sample, *x*_*knn*_ represents the *k* nearest neighbors, and α is the randomly selected point.

#### Data rescaling

Before applying ML algorithms, one important step required in data preprocessing is data rescaling. This makes the various ML models more effective. The dataset contained various scales for various quantities (e.g., age, mean arterial pressure, heart rate, C-reactive protein, and procalcitonin). Therefore, we perform data normalization to rescale all real-valued features:


$$\begin{array}{l} \tilde{x}=\frac{x-avg}{std}, \end{array}$$

where *x* denotes the value, *avg* is the average of the values, and *std* is the standard deviation. For models like logistic regression, which rely on the magnitude of values to determine coefficients, this step is highly important.

#### Feature selection

Some features contribute to predicting a variable of interest than others. Feature selection is, therefore, performed to automatically select those features. By doing this, the accuracy is improved, overfitting is reduced, and most importantly, the time required for training is reduced. Irrelevant features can reduce the performance of several machine learning models. We investigated six techniques of feature selection: Lower variance, L1 regularization-based feature selection, L2 regularization-based feature selection, Univariate feature selection, Tree-based feature selection, and Principal Component Analysis (PCA).

- Eliminate lower variance (LV): Variance quantifies how widely apart a collection of data is. When the variance is 0, all of the data values are the same and vice-versa. The formula to compute variance is given as

$$\begin{array}{l} \sigma^{2}=\frac{1}{n}\overset{n}{\underset{i=1}{\sum }}{\left(x_{i}-\bar{x}\right)}^{2} \end{array}$$

where *n* is the number of pieces of data, *x*_*i*_ is each of the values in the data, and $\overset{\bar{}}{x}$ is the mean (average) of the data. If the variance is low or near zero, that feature is relatively constant and will not increase the model's performance. Hence, it should be removed.

- Univariate feature selection: The univariate feature selection (UFS) selects the best features by applying univariate statistical tests. Specifically, UFS examines each feature exclusively to determine the strength of the feature's relationship with the response variable using the Chi-Squared Test. Given the data of two variables, the Chi-Squared Test observes count *O* and expected count *E*. Chi-Square measures how expected count *E* and observed count *O* deviate from each other. The formula for chi-square is

$$\begin{array}{l} x_{2}^{C}=\;\sum \frac{{\left(O_{i}-E_{i}\right)}^{2}}{E_{i}} \end{array}$$

where *c* is the degree of freedom, *O* denotes observed values(s), and *E* denotes expected values(s).

- L1/L2 regularization-based feature selection: The solutions to linear models penalized with the L1 norm are sparse: many estimated coefficients are 0. L1/L2 regularization-based feature selection can reduce the dimensionality of the data by selecting features with non-zero coefficients. The L2 norm adds “squared magnitude” of coefficient as a penalty term to the loss function as

$$\begin{array}{l} \overset{n}{\underset{i=1}{\sum }}{\left(y_{i}-\overset{p}{\underset{j=1}{\sum }}x_{ij}\beta_{j}\right)}^{2}+\lambda \overset{p}{\underset{j=1}{\sum }}\beta_{j}^{2} \end{array}$$

Whiles the L1 norm adds an absolute value of the magnitude of coefficient as a penalty term to the loss function as


$$\begin{array}{l} \overset{n}{\underset{i=1}{\sum }}{\left(y_{i}-\overset{p}{\underset{j=1}{\sum }}x_{ij}\beta_{j}\right)}^{2}+\lambda \overset{p}{\underset{j=1}{\sum }}|\beta_{j}| \end{array}$$

- Tree-based feature selection: We used tree-based estimators to calculate the impurity-based feature importance; this can be used to remove irrelevant features. We used a Random Forest algorithm. We selected 50 decision trees, each constructed using a random extraction of observations from the dataset and features. Because most data characteristics are not seen by some trees, they (the tress) are de-correlated which makes them less prone to over-fitting. Each tree is also a series of yes-no questions based on a single or many characteristics. The tree splits the dataset into two buckets at each node, each containing more similar observations and distinct from those in the other bucket. As a result, the significance of each attribute is determined by how “pure” each of the buckets is.
- Principal Component Analysis (PCA): We utilized PCA to reduce the dimensions of our larger dataset. Essentially, the reduced dataset still contains much of the information in the large set. It is accomplished by evaluating the correlation between features in order to find the most important principal components. Although it is clear that there are other better options such as t-SNE and UMAP for dimension reduction, these reasons were considered before choosing PCA for this task. t-SNE involves a lot of calculations and computations because it computes pairwise conditional probabilities for each data point and tries to minimize the sum of the difference of the probabilities in higher and lower dimensions. t-SNE has a quadratic time and space complexity in the number of data points. This makes it particularly slow, computationally quite heavy and resource draining. Also, the main disadvantage of UMAP is its lack of maturity. It is a very new technique, so the libraries and best practices are not yet firmly established or robust. The short summary is that PCA is far and away from the fastest option, it is deterministic and linear. However, we potentially gave up a lot for that speed. We set the principal components to 26.

### Experimental setup

We perform machine-learning analysis on 535 different patients, each with 45 features. Since it is a binary problem, we categorized each patient into one class at a time. We designed three experiments to evaluate the models: (1) feature selection techniques to select appropriate features for the models, (2) experiments on the imbalanced original dataset, and (3) experiments on the balanced data *via* SMOTE.

### Prediction models

We compared several models to evaluate their effectiveness in predicting pneumonia: Logistic Regression (LR), Naïve Bayes (NB), Support Vector Machine (SVM), Adaboost Decision Tree (ADT), K-Nearest Neighbor (KNN), Random Forest (RF), Extreme Gradient Boosting (XGBoost), and Multilayer Perceptron (MLP). These models have been extensively used for many classification tasks.

### Evaluation metrics

**Figure 1:**
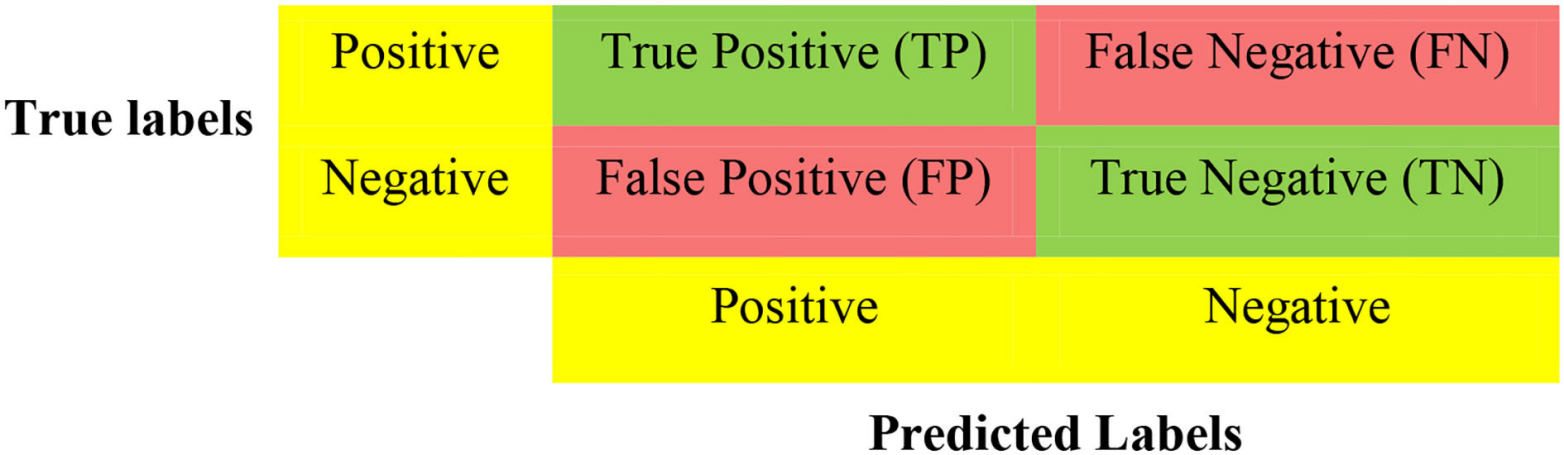
Confusion matrix.

Following previous works (16, 17), and considering that machine learning models have multiple tuning parameters, which are essential for model performance, we adopted 5-fold cross-validation (CV) to evaluate all the classification models using confusion matrices (Figure 1) and ROCs. CV is a resampling technique used for evaluating and validating ML algorithms based on a small dataset sample. The dataset is randomly divided into k equal portions (number of folds). In training the model, the residual k-1 dataset is used, while the remaining dataset (validation dataset) is used to test the model. The CV procedure is then replicated k times with different folds as the test set each time. In order to achieve a specific outcome, all k outcomes from k-folds are summed and the average is then calculated (18, 19). In the 5-fold cross-validation, we randomly partition the dataset into five equal subsamples. One subsample was used as the validation set and the remaining four subsamples were used as the training set. We divided all datasets into 80% training and 20% testing. We used the training data during the feature selection and training. However, the test data was used for model selection.

For binary classification, multiple criteria are needed in evaluating the performance of the models. As such, we evaluate the performance of the various models based on f-measure, Area Under the Curve of the Receiver Operator Characteristic (AUC-ROC), accuracy, recall, and precision. These performance metrics can be determined using True Positives (TP), True Negatives (TN), False Positives (FP), and False Negatives (FN) as seen in Figure 1. The accuracy is the proportion of all correctly predicted samples to the total samples. The recall rate is the proportion of positive samples correctly identified as positive to the total number of positive samples. This metric is critical for our work since prediction models want to identify as many positive samples as possible. The precision defines the ratio of the number of positive samples accurately predicted as positive to the number of positive examples. Naturally, an excellent predictive model seeks a high recall rate and precision. There is, however, a trade-off between recall rate and accuracy; the F-measure provides a thorough assessment by computing the harmonic mean of recall and precision. Table 1 shows the equations used for calculating the desired performance metrics: accuracy, precision, recall, and f-measure.

**Table 1:** Performance evaluation metrics equations.

**Metric**	**Equation**
Accuracy	$\frac{TP+TN}{TP+FP+FN+TN}$
Recall	$\frac{TP}{TP+FN}$
Precision	$\frac{TP}{TP+FP}$
F-measure	$2\times \frac{Recall\times Precision}{Recall+Precision}$

## Results

### Data balancing, rescaling, and feature selection

**Figure 2:**
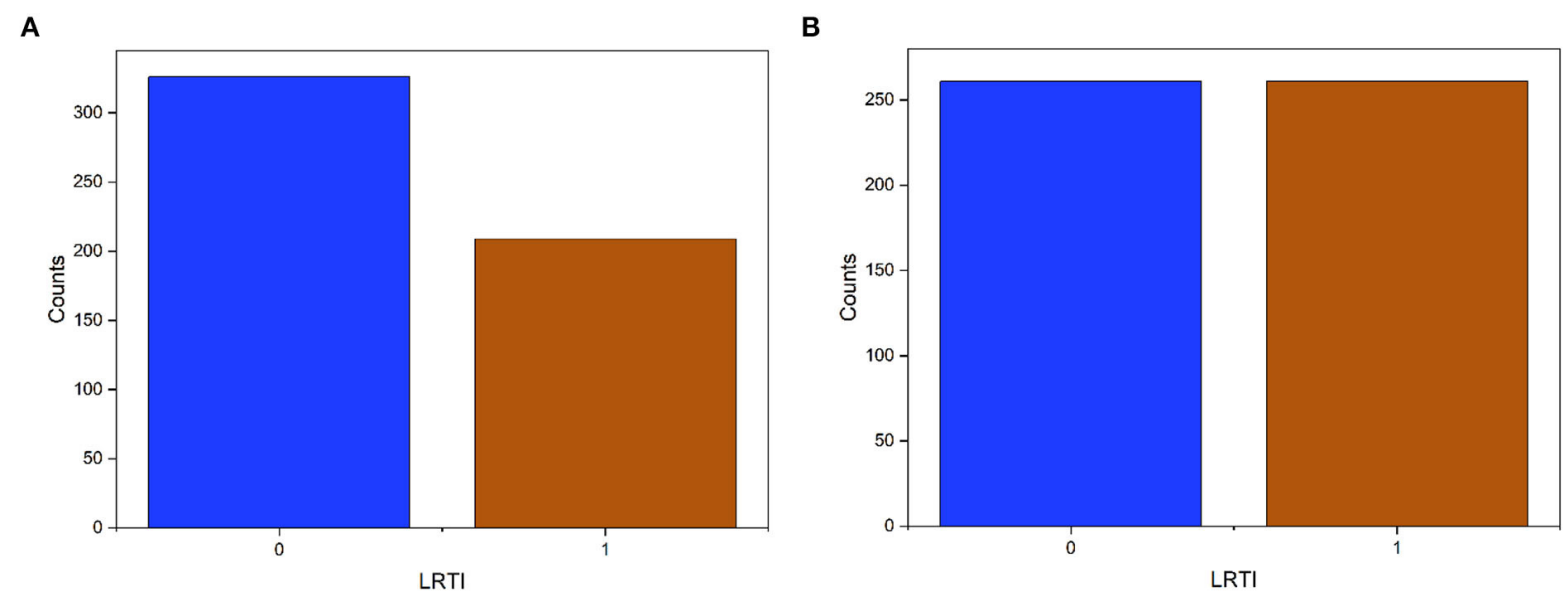
Target data (LRTI) distribution before and after applying SMOTE. The label '0' is pneumonia and “1” for bronchitis. **(A)** Imbalanced data. **(B)** Balance data.

The dominant class of the dataset had 22% more samples (Figure 2). After SMOTE, we obtain two types of datasets: the original imbalanced dataset and the SMOTE dataset.

We then used Logistic Regression as the baseline model to choose the appropriate feature selection methods. The results show that the Tree-based is most effective on the original data followed by UFS (Table 2). In the SMOTE dataset, PCA is most effective, followed by LV (Table 3). We used Tree-based and UFS in subsequent experiments on the original dataset and reported the best results. Likewise, we used PCA and LV in subsequent experiments on the balanced SMOTE dataset and reported the best results.

**Table 2:** LR prediction result of feature selection methods on original dataset.

**Feature selection**	**Accuracy**	**Precision**	**Recall**	**F1-score**
LV	80.4	83.7	84.4	84.0
UFS	82.6	85.8	85.9	85.8
L1	75.9	79.0	82.5	80.7
L2	77.9	82.3	81.6	81.8
Tree-based	83.0	85.7	86.8	86.2
PCA	81.1	84.5	84.7	84.6

**Table 3:** LR prediction result of feature selection method on balanced dataset.

**Feature selection**	**Accuracy**	**Precision**	**Recall**	**F1-score**
LV	83.6	85.4	81.3	83.4
UFS	82.2	83.3	80.9	82.0
L1	77.3	78.2	75.4	77.1
L2	79.1	81.5	75.2	78.0
Tree-based	82.0	83.1	80.3	81.6
PCA	85.4	86.6	83.0	84.7

### Evaluation of the performance of the machine learning models on the original dataset

We conducted experiments to acquire empirical evidence on the original imbalanced dataset using the ML models listed above. From Table 4, the Ensemble machine learning models such as RF (accuracy = 92.0%, precision = 91.3%, recall = 96.0%, f1-Score = 93.6%) and XGBoost (accuracy = 90.8%, precision = 92.6%, recall = 92.3%, f1-score = 92.4%) achieved the highest performance accuracy while NB achieved the lowest performance accuracy on the original imbalanced dataset. Also, ADT (accuracy = 90.1%, precision = 91.3%, recall = 92.7%, F1-Score = 91.9%) had a performance which was almost similar to that of XGBoost.

**Table 4:** Machine learning model prediction results on the original dataset.

**Model**	**Accuracy**	**Precision**	**Recall**	**F1-score**
LR	81.4	82.7	84.2	84.3
NB	59.8	89.6	39.2	53.7
SVM	80.7	82.8	86.5	84.5
ADT	90.1	91.3	92.7	91.9
KNN	72.1	87.3	63.8	73.5
RF	92.0	91.3	96.0	93.6
XGBoost	90.8	92.6	92.3	92.4
MLP	79.4	83.7	82.5	82.9

**Figure 3:**
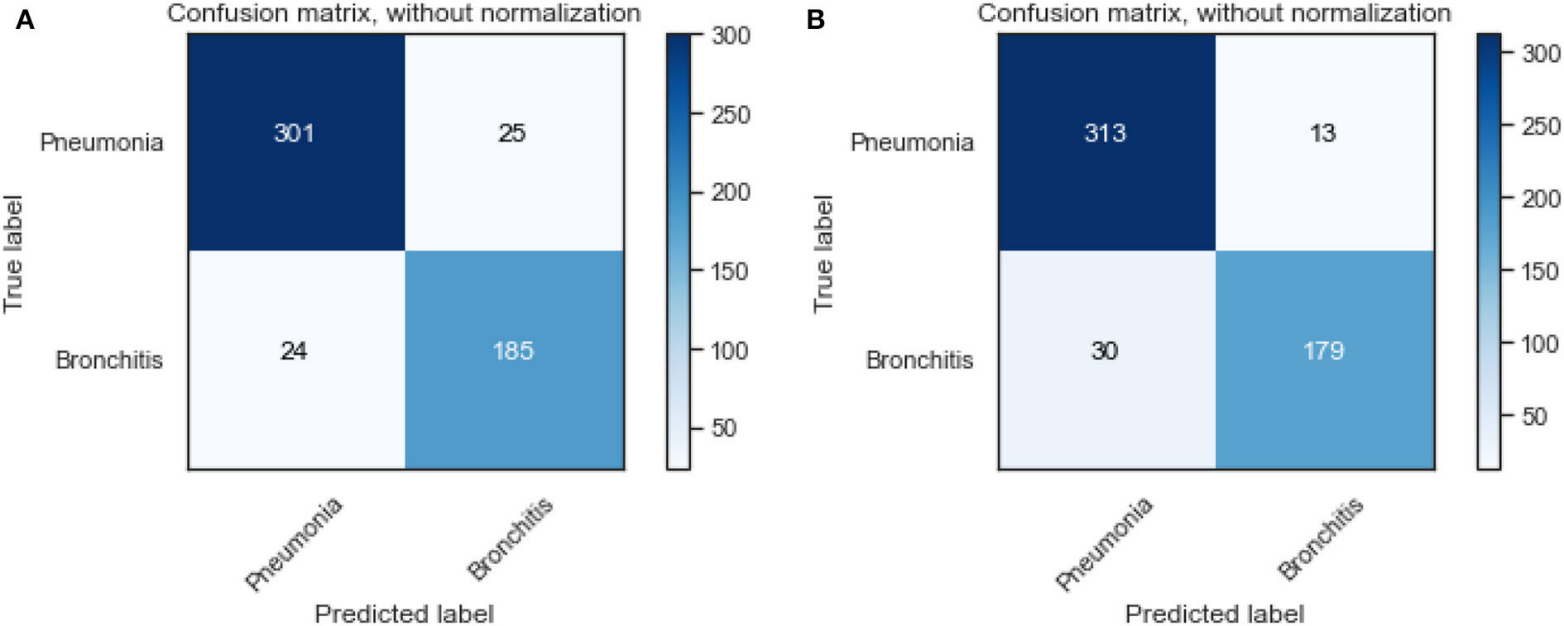
Confusion matrix of XGBoost and random forest on the original dataset. **(A)** XGBoost. **(B)** RF.

We also visualize the confusion matrix of RF and XGBoost in Figure 3. We observe that the XGBoost model wrongly predicted more pneumonia samples (25) than the RF model (13). However, XGBoost performed better than RF when predicting other LRTIs other than pneumonia. Generally, it can be deduced that the models could learn from the data.

**Figure 4:**
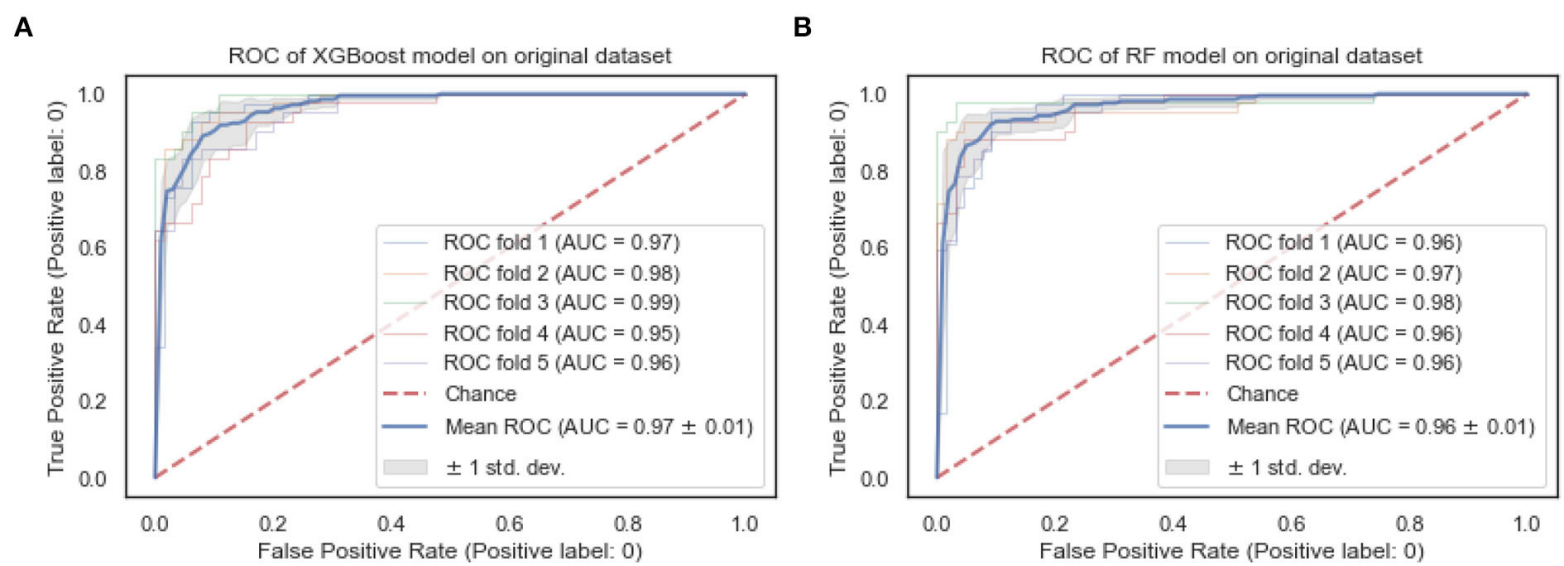
ROC curves of XGBoost and random forest on the original dataset. **(A)** XGBoost. **(B)** RF.

The ROC curves of the XGBoost and RF are shown in Figure 4. We observe that both the XGBoost and RF models achieve a similar performance of 0.97 and 0.96, respectively. Also, the “steepness” of the ROC shows that the XGBoost model has a slightly high positive rate than the RF model.

**Figure 5:**
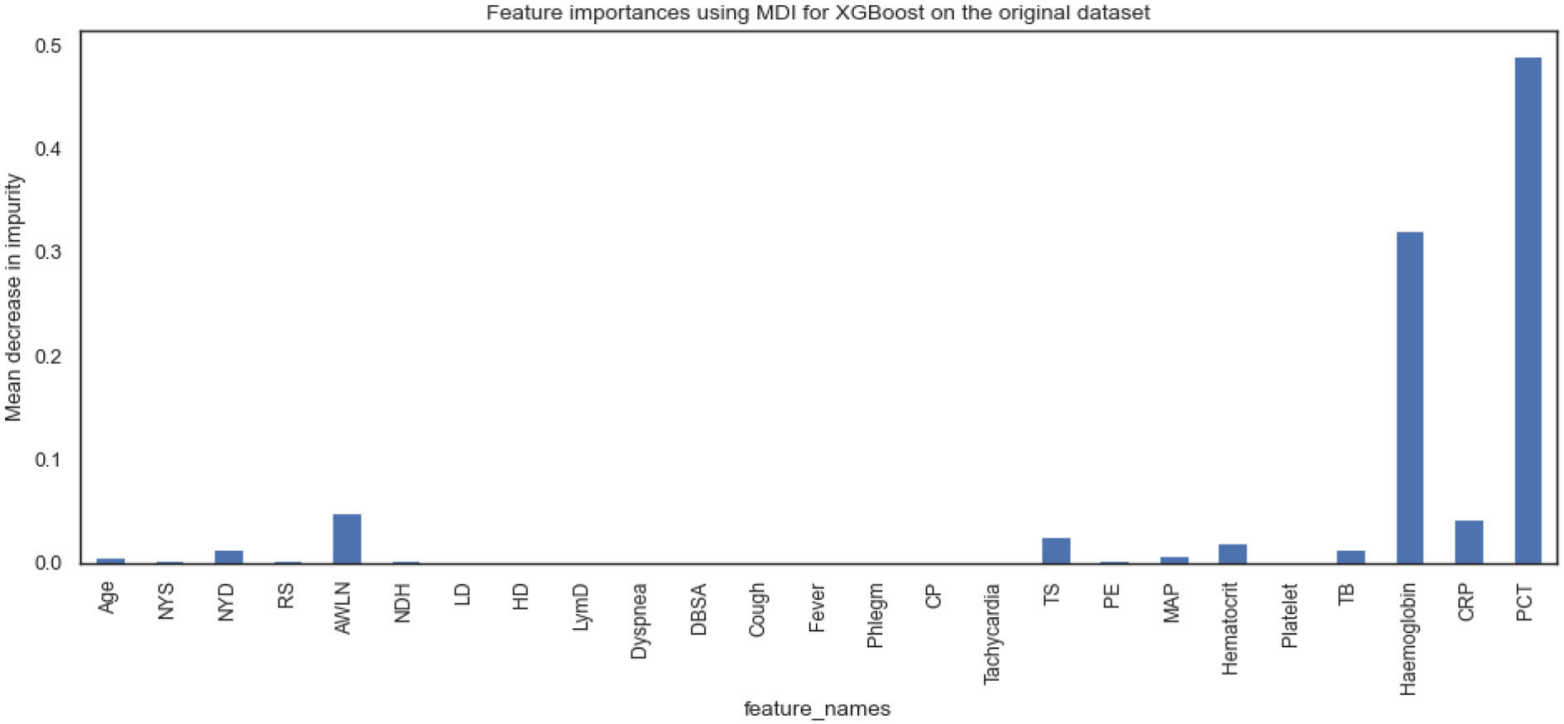
Feature importance according to XGBoost model on the original dataset.

**Figure 6:**
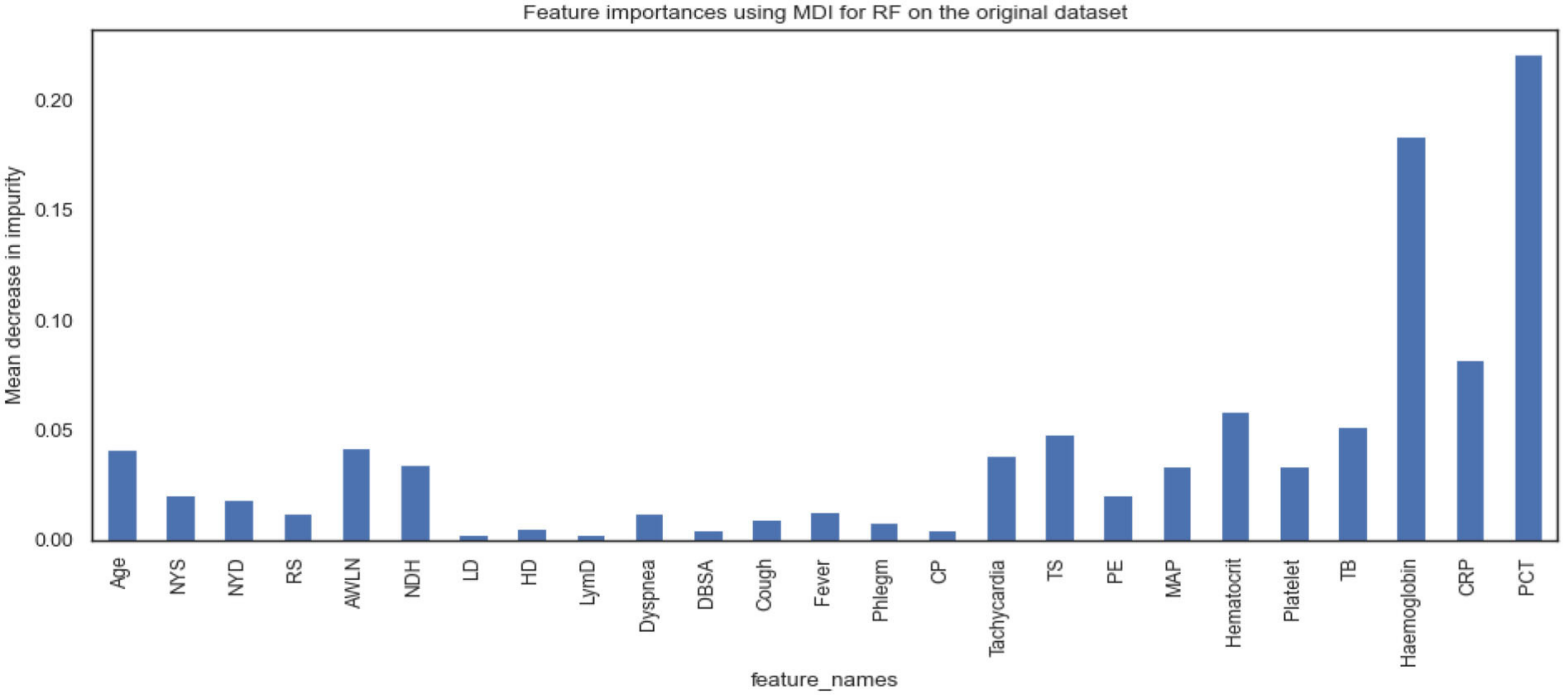
Feature importance according to the RF model on the original dataset.

Figures 5, 6 show the essential features that XGBoost and RF models consider essential for prediction. Both XGBoost and RF models consider hemoglobin, C-reactive protein, and procalcitonin features very notably. Tracheal secretion, antibiotics taken within the last 90 days, total bilirubin and hematocrit features are also considered necessary by both models, but their importance is relatively low compared with those listed earlier. However, XGBoost does not consider some features necessary (e.g., age, years of smoking, years of drinking, dyspnea, tachycardia) compared to the RF model.

### Evaluation performance of the machine learning models on the SMOTE dataset

We also conducted experiments to acquire empirical evidence on the SMOTE dataset using similar machine learning models listed above.

From Table 5, the RF model achieved the highest performance followed by XGBoost and ADT, while NB achieved the lowest prediction performance. The f1-scores of RF and XGBoost are 92.0 and 91.2%, respectively, which indicates how robust the models are.

**Table 5:** Machine learning model prediction results in the balanced dataset.

**Model**	**Accuracy**	**Precision**	**Recall**	**F1-score**
LR	83.6	84.9	81.2	83.1
NB	68.4	75.8	54.4	62.7
SVM	81.1	83.0	77.2	80.1
ADT	91.0	91.2	90.1	90.9
KNN	75.0	91.9	54.8	68.4
RF	92.2	93.0	91.2	92.0
XGBoost	91.2	91.1	91.6	91.2
MLP	81.4	81.9	83.2	82.4

**Figure 7:**
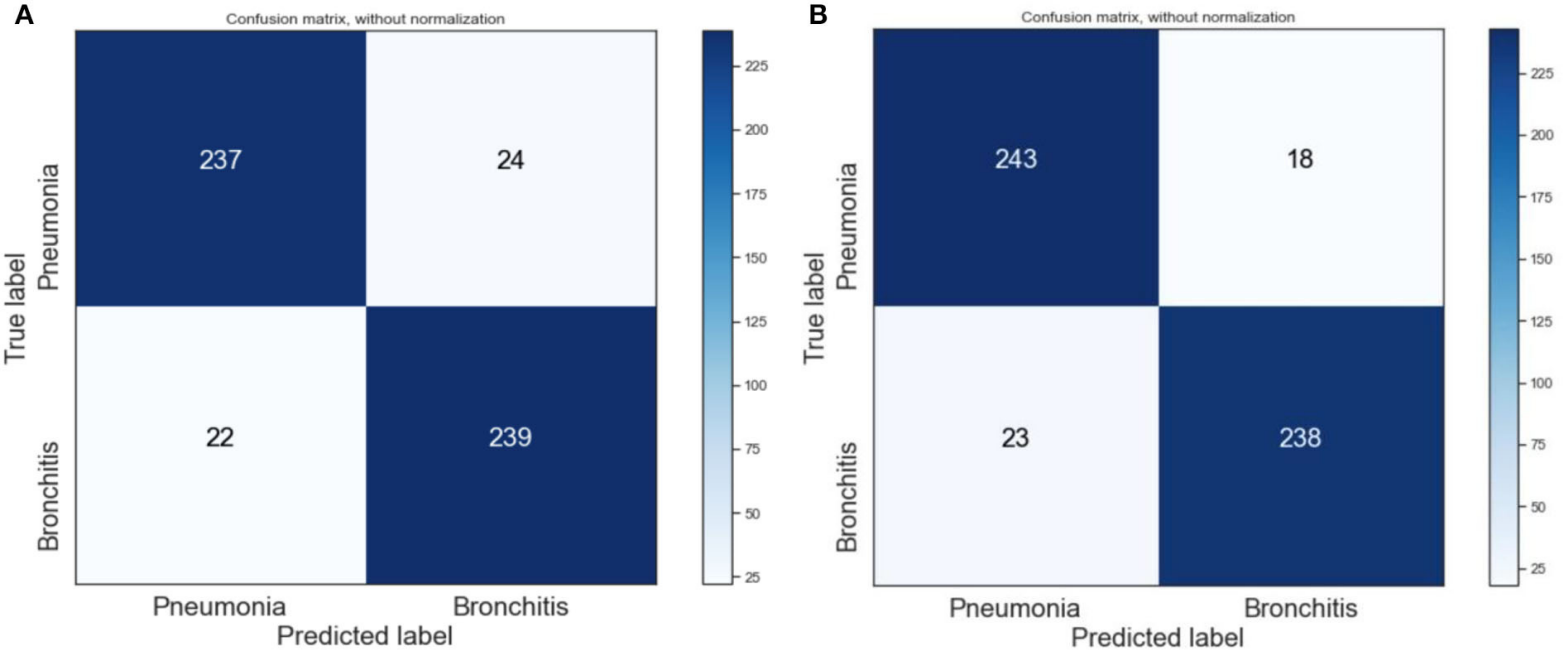
Confusion matrix of XGBoost and RF on SMOTE data. **(A)** XGBoost. **(B)** RF.

We also visualized the confusion matrix of XGBoost and RF in Figure 7 and made the following observations. The XGBoost model wrongly predicted more pneumonia samples (24) than the RF model (18). Generally, it was observed that the models could learn significantly from the data.

**Figure 8:**
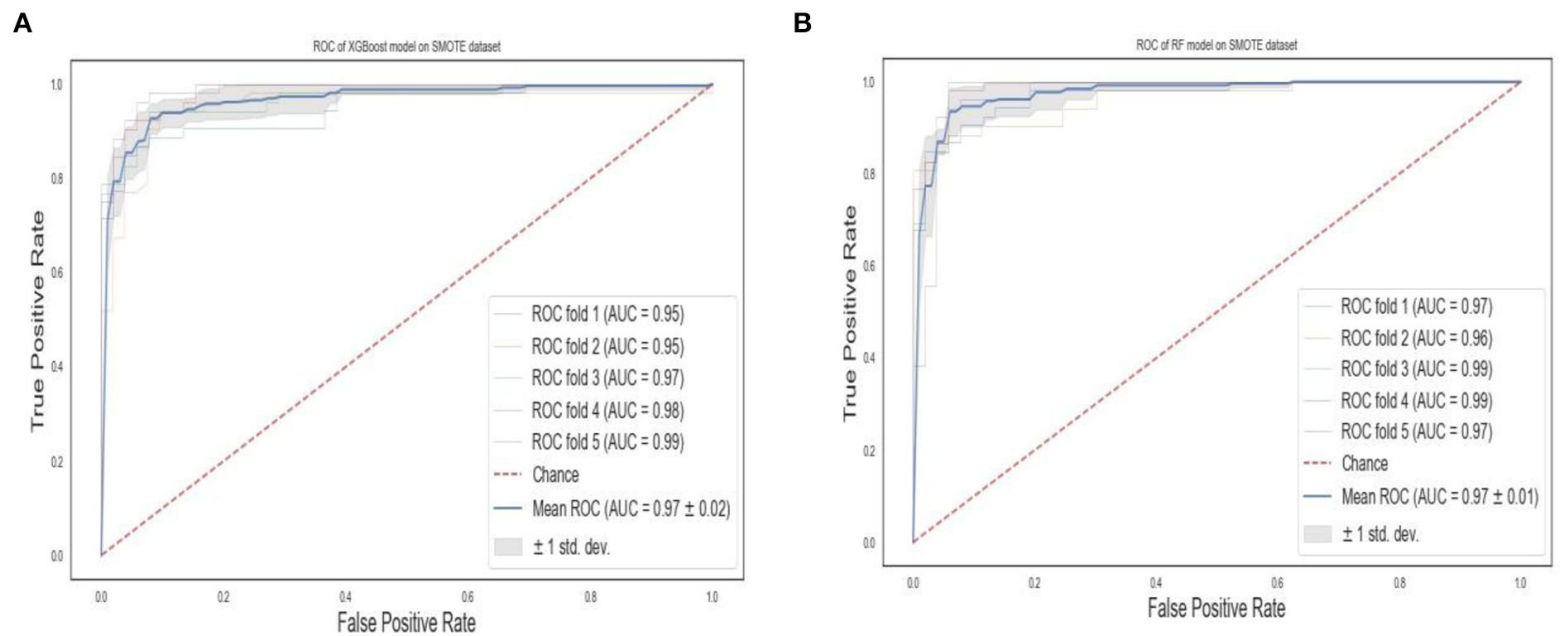
ROC curves of XGBoost and random forest on the SMOTE dataset. **(A)** XGBoost. **(B)** RF.

The ROC curves of the XGBoost and RF are shown in Figure 8. We observe that RF models achieve the same superior performance as the XGBoost model. Also, the “steepness” of the ROC shows that the RF model has a slightly high positive rate than the XGBoost model.

**Figure 9:**
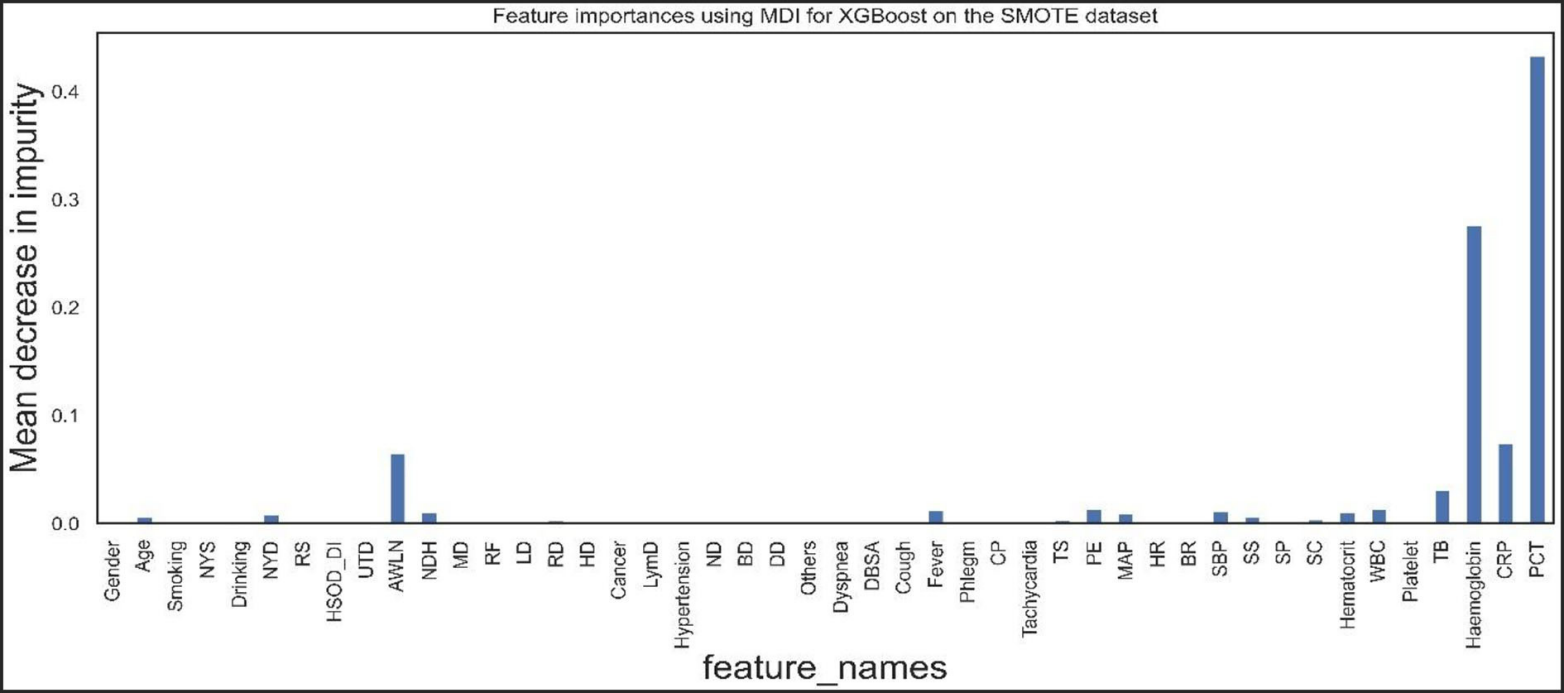
Feature importance according to the XGBoost model on the SMOTE dataset.

**Figure 10:**
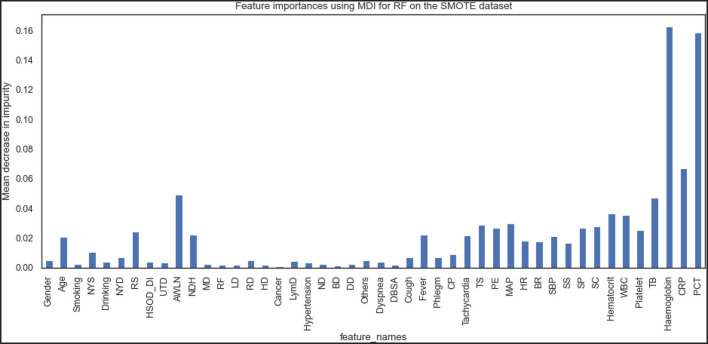
Feature importance according to the RF model on the SMOTE dataset.

Figures 9, 10 show the features the XGBoost and RF model considers vital for prediction. XGBoost and RF models consider hemoglobin, hematocrit, drinking, mean arterial pressure, plural effusion, tracheal secretion, tachycardia, years of smoking, C-reactive protein, antibiotics taken within the last 90 days, procalcitonin, and total bilirubin features significantly in the prediction.

Because we performed machine learning experiments on both the original and the SMOTE data, we run ANOVA to compare whether there are statistical differences in the prediction performances of the models before and after SMOTE. We did this by comparing their AUCs. AUC is a measure of the accuracy of a quantitative diagnostic test. It is the average value of specificity for all values of sensitivity. Table 6 shows the AUCs of the models for the original and balanced datasets. We observed that the AUCs of some models (LR, MLP, KNN, NB) differ significantly in the two datasets while others (SVM, XGBoost, ADT, RF) achieved similar or showed no significant difference in their before and after SMOTE AUCs.

**Table 6:** AUCs of the various models before and after SMOTE.

**Model**	**Original dataset**	**Balanced dataset**	***P* value**
LR	89	91	0.032
NB	82	76	0.019
SVM	89	86	0.221
ADT	91	94	0.071
KNN	79	84	0.016
RF	96	97	0.050
XGBoost	97	97	0.314
MLP	80	86	0.005

### Decision boundaries of the models

**Figure 11:**
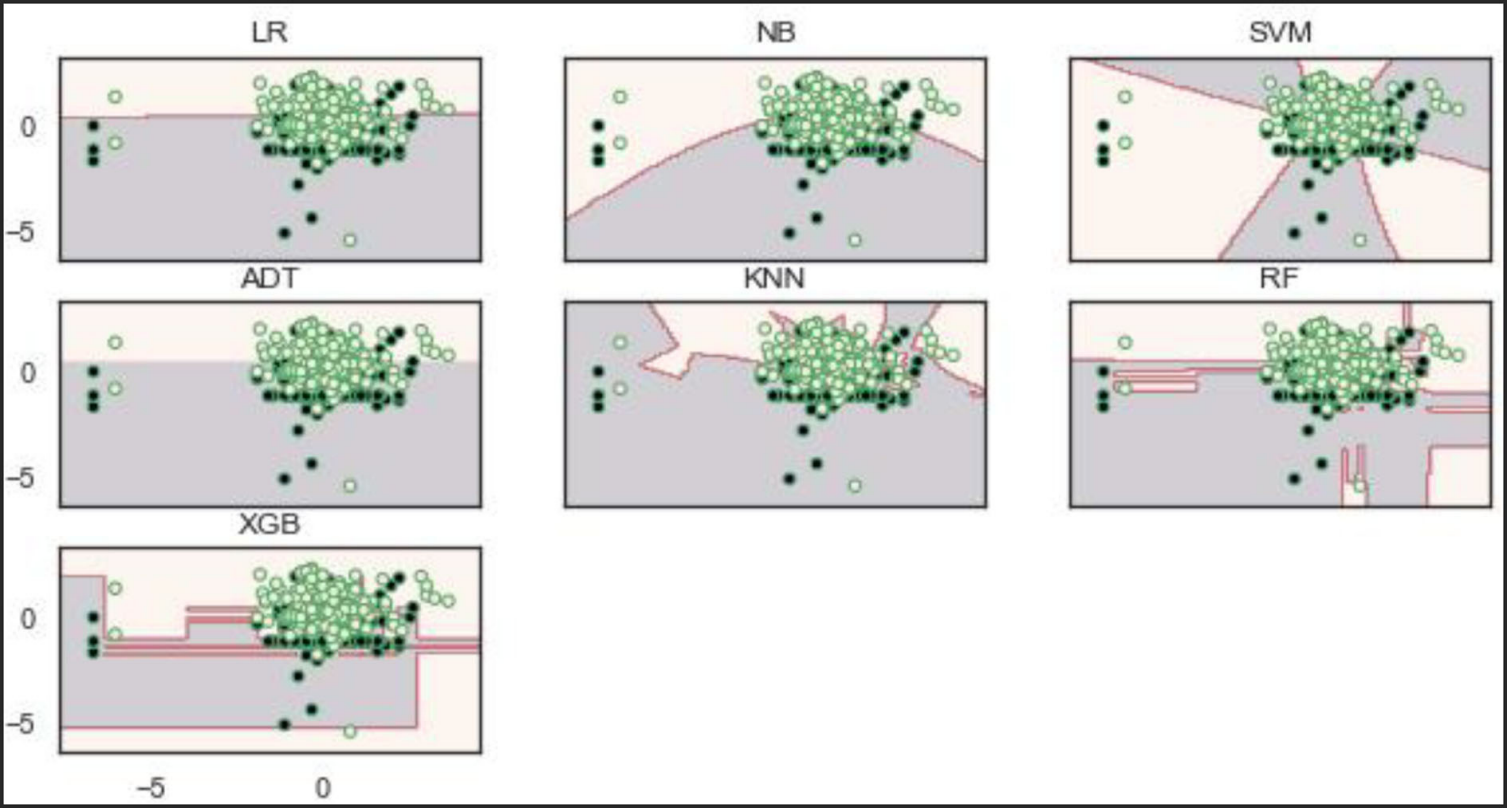
Decision boundaries of the models on the original dataset.

Decisions, or boundaries, are lines drawn using the best decisions (for our purposes, binary classifiers) that separate samples of one class from the other class. All instances of one class and the opposing class are found on each side. The decision boundaries of the models show that the RF and XGBoost models learn a robust decision boundary (Figure 11). RF and XGBoost models can learn and correctly classify the samples at the bottom compared to the other models. This observation is expected because the two models (RF and XGBoost) achieved the best performance on the original dataset.

**Figure 12:**
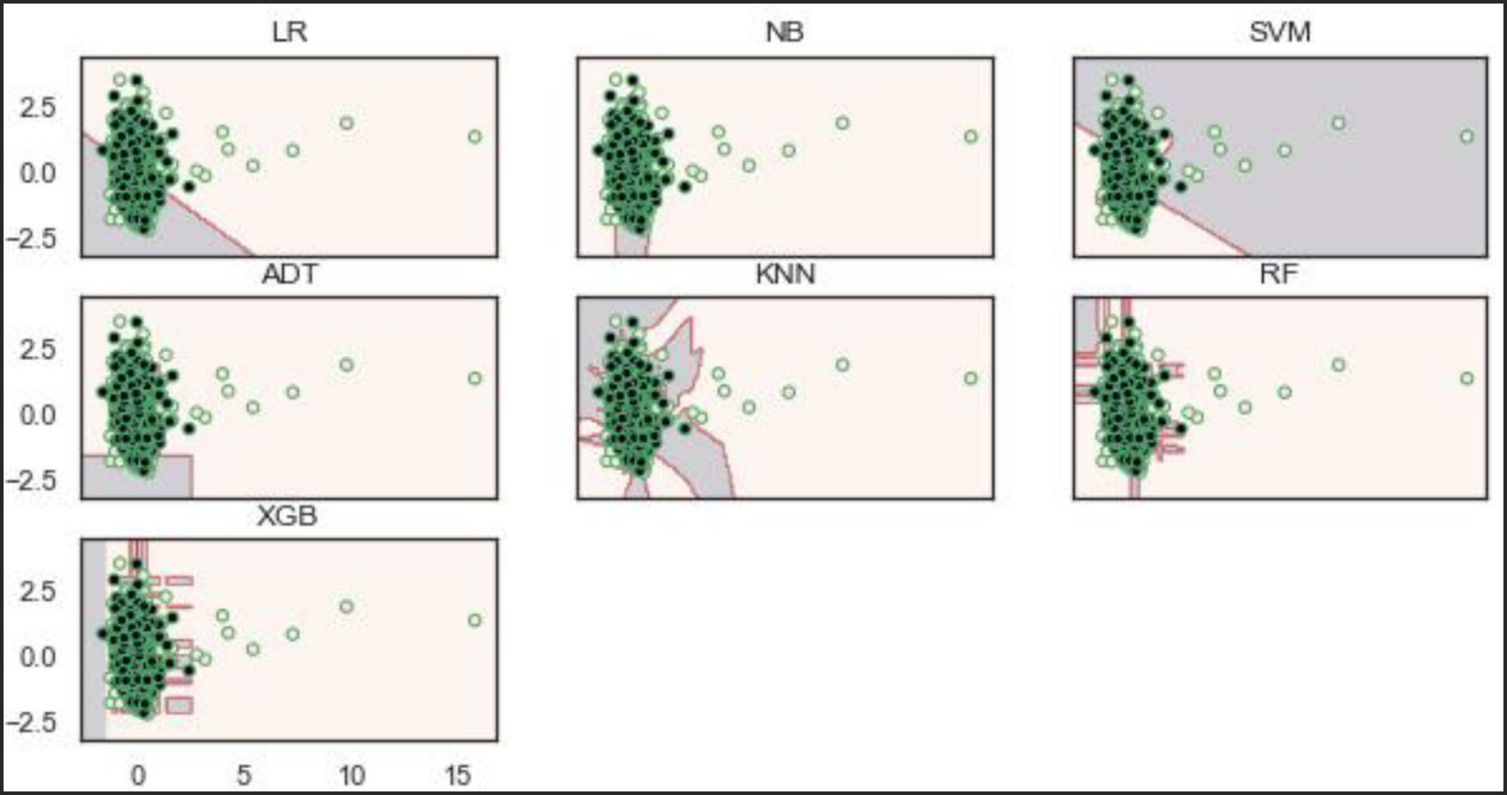
Decision boundaries of the models on the balanced dataset.

Based on the balanced dataset (Figure 12), the ADT, RF, and XGBoost models demonstrate a well-bodied boundary while LR and SVM show poor boundaries.

### External validation of the models

**Figure 13:**
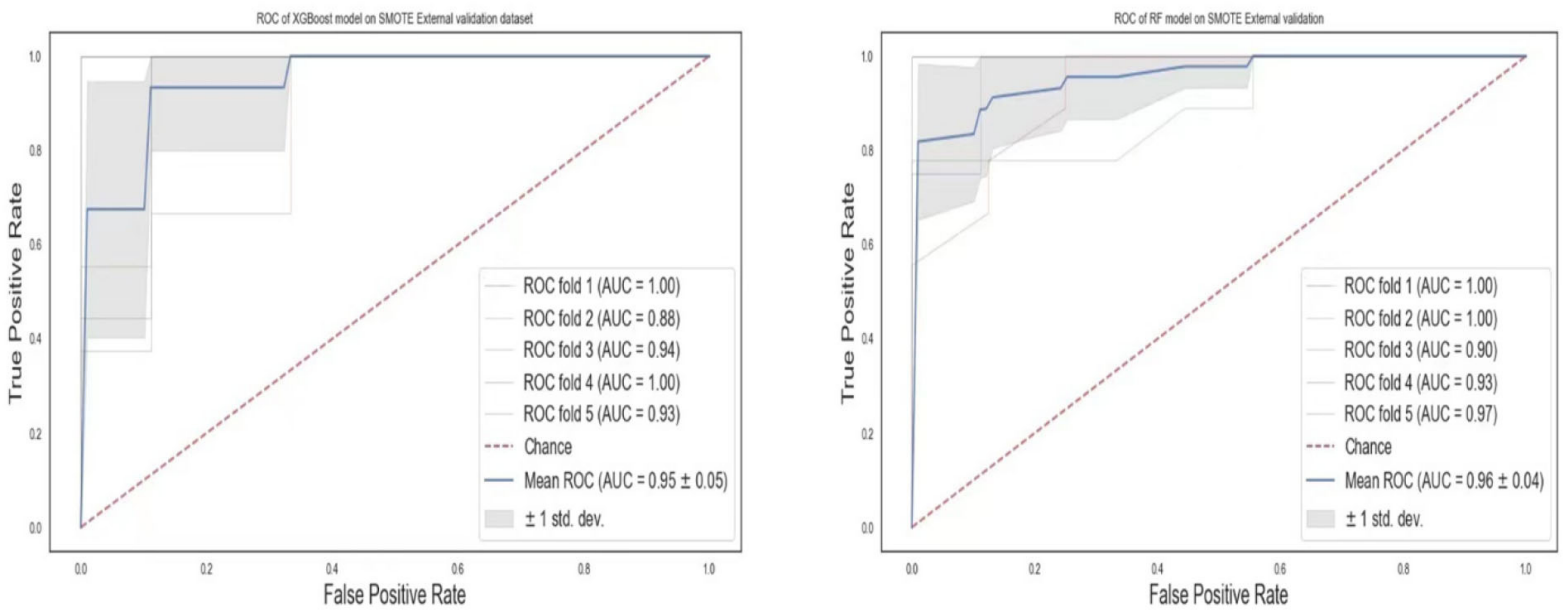
AUROC curves for the external validation dataset. **(A)** XGBoost. **(B)** RF.

To validate our models for generalizability, we externally collected data from 77 patients with lower respiratory tract infections (either pneumonia or bronchitis). The two best models, RF and XGBoost, were chosen for the external validation. Although the data used for this experiment was limited, the models were still robust in the prediction of pneumonia (Table 7). The ROCs values (Figure 13) show AUCs of 95 and 96% for XGBoost and RF models confirming that our models have good generality.

**Table 7:** External validation results from the best models.

**Model**	**Accuracy**	**Precision**	**Recall**	**F1-score**
RF	88.6	84.8	95.6	89.7
XGBoost	88.7	86.4	93.1	89.3

## Discussion

Laboratory tests, blood culture, C-reactive protein, serology, and procalcitonin are diagnostic tests with varying rates of accuracy (20). Our models showed that individual clinical history and symptoms do not have adequate discriminatory power except dyspnea, diminishing breath sound on auscultation, cough, fever, and phlegm to diagnose pneumonia. Earlier studies have shown that radiographic pneumonia cannot be diagnosed by a single clinical symptom and this was consistent with our study (21). Fever, tachycardia, and breathing rate were among the most useful predictors of the clinical signs. Evidence suggests that adults whose respiration rates exceed 20 breaths per minute are probably unwell, and those whose respiration rates exceed 24 breaths per minute are deemed to be critically ill (22). The findings of this study are similar to previously published study (23). Similar to other studies (24), diminishing sound on auscultation was shown to be an important predictor of pneumonia in our models. As part of externally validated prediction models for pneumonia, diminishing sound on auscultation, tachycardia, and fever were found to be useful predictors (25). In a study by Niederman et al., it was postulated that patients with symptoms such as cough, sputum production, and/or dyspnea, in addition to other indicators like fever and auscultatory findings of abnormal breath sounds may have a higher risk of developing pneumonia (26). Tracheal secretion, antibiotics taken within the last 90 days, total bilirubin, and hematocrit were all features considered important for pneumonia prediction in our models. Tracheal secretion has been noted by several authors as an important diagnostic tool for pneumonia (27, 28).

Biomarkers can support clinicians in the differentiation of bacterial pneumonia from other disorders. Among all the variables tested in our prediction models, biomarkers such as CRP and PCT demonstrated the most significant discriminating power in the prediction of pneumonia. CRP and PCT, are extensively used in the monitoring of treatment of severe infections in the ICU. PCT is a marker that is strongly correlated with bacteria load and severity of infection (29). Also, a high PCT level indicates a bacterial infection rather than a viral infection. A meta-analysis reported that the use of PCT to guide antibiotic treatment in pneumonia resulted in a reduction in exposure to antibiotics (30). A PCT level of >0.25 ng/ml is indicative of an underlying bacterial infection (31). This evidence supports our results that, PCT can accurately predict pneumonia. Among patients with pneumonia, the prognostic value of PCT and its correlation with disease severity has been exclusively studied (31). In ambulatory care, CRP has been widely used as a point of care test. Researchers have examined CRP as a diagnostic tool in screening for inflammation and detecting bacterial infections (32). Through the use of CRP in primary care, antibiotic exposure can be reduced in suspected LRTI (risk ratio [RR] 0·78 [95% CI 0·66–0·92]) (33). According to the NICE's guidelines, antibiotics should not be given to patients without a convincing clinical diagnosis of pneumonia, when their CRP is <20 mg/L (34). Our results showed that CRP is a useful diagnostic tool to predict pneumonia. This finding is similar to previous studies (32). CRP has been shown to improve the diagnostic discriminatory power of models built on basic signs and symptoms during the prediction of patients with pneumonia (35).

From our machine learning models, RF and XGBoost were considered the best models on both the original dataset and the SMOTE balanced data. RF model has demonstrated superiority and stability in numerous medical studies (36, 37). Because of the extensive application of integrated algorithms, the RF model has become a well-established technology (38). RF uses the bagging ensemble technique for classification. Decision trees (DTs) are the building blocks of the RF classifier. In order to train uncorrelated decision trees, each tree is trained with a random sample selected from the dataset. Then, final decisions are made by combining the outputs from all the DTs. Because the forest is randomized, it slightly increases the biasness of the forest. However, due to the averaging of the outputs, its variance decreases, hence yielding an overall better model. As an efficient and scalable tree boosting system (39), the XGBoost model has shown excellent performance in several ML competitions, primarily due to its simplicity and accuracy in prediction (40). Our study showed that the XGBoost model had a good performance, with an F1-score of 92.4% and an accuracy of 90.8%. Because ensemble ML models (RF and XGBoost) integrate multiple base learners or classifiers, they are robust and have high accuracy which was confirmed in this study. All models on the original data had AUC values lower than those observed in the ensemble ML models. However, comparing XGBoost, a boosting ensemble method to RF which is a bagging ensemble method, RF needs to train a large amount of decision trees and aggregate them, thereby requiring longer time to trade numerous random computations for high accuracy. Moreover, XGBoost leverages second order derivative and implements sampling method in each iteration to alleviate overfitting and speed up computation. In addition to the RF and XGBoost models, ADT also achieved better performance on the SMOTE balanced data. The strength of AdaBoost resides in combining weak learners with a powerful learner with a high prediction accuracy based on the adjustments of weights (41). These weights are mainly related to samples that are used by the learner in the training phase. The learners in this phase can generate a set of misclassified samples. AdaBoost tries to resolve this issue by providing appropriate weights for samples that have been wrongly classified. Those samples that are misclassified are assigned a larger weight while samples that are already well classified receive a smaller weight. The unique ability of AdaBoost to spot the misclassified samples, correct them, and re-feed them to the next learner until an accurate predictor model is constructed, makes it one of the best powerful binary classification models. Comparing the results of this study with other studies that used non-invasive measure to build algorithms for disease predictions, we realized that our results were comparable to these studies or even performed better than most studies (Table 8).

**Table 8:** Comparing prediction performance from various studies that used non-invasive measures.

**Models**	**Predicted disease**	**Performance evaluation**	**Ref**
DT, SVM, LR	Pneumonia	Accuracy-84, 82, 83	(42)
RF, LightGBM, SVM, DT	COVID-19	Accuracy-89, 88, 84, 82	(43)
LogitBoost, RF, DT	Blood diseases	Accuracy-98.2, 97.1, 97	(44)
XGBoost, LightGBM		Accuracy-93, 91	(45)
LR	COVID-19	Specifificity-0.95; AUC-0.971; Sensitivity-0.82	(46)
RF, XGBoost	Pneumonia	Accuracy-92, 90.8; AUCs-0.96, 0.97	This study

## Conclusions

This study predicted pneumonia from other LRTIs such as bronchitis using biomarkers, physical indicators, and laboratory parameters. Individual clinical history and symptoms do not have adequate discriminatory power, hence should not be considered in unison during the diagnosis of pneumonia. Two biomarkers, C-reactive protein and procalcitonin, in addition to other features, were considered very important in the prediction of pneumonia. Compared to the SMOTE balanced data, using the original data achieved a higher prediction performance. Therefore, we can conclude that the original dataset was sufficient to predict pneumonia without balancing. RF and XGBoost were considered the best models on both the original dataset and the SMOTE balanced data. From this, we can conclude that the ensemble ML models performed better in the prediction of pneumonia.

## Data availability statement

The raw data supporting the conclusions of this article will be made available by the authors, without undue reservation.

## Author contributions

CE contributed to the conceptualization, study design, data collection, interpretation, and writing of manuscript. RM, ED, RQ, and CA contributed to data collection, literature search, data analysis, and interpretation. YoW contributed to the conceptualization, data analysis, interpretation, writing of the manuscript, fund sourcing, and supervision. LM and YaW contributed to data analysis, interpretation, writing of the manuscript, and supervision. All authors contributed to the article and approved the submitted version.

## Funding

This work was supported by the National Natural Science Foundation of China (No. 81973099).

## Conflict of interest

Author YaW was employed by Center of Health Management, General Hospital of Anyang Iron and Steel Group Co., Ltd. The remaining authors declare that the research was conducted in the absence of any commercial or financial relationships that could be construed as a potential conflict of interest.

## Publisher's note

All claims expressed in this article are solely those of the authors and do not necessarily represent those of their affiliated organizations, or those of the publisher, the editors and the reviewers. Any product that may be evaluated in this article, or claim that may be made by its manufacturer, is not guaranteed or endorsed by the publisher.
